# Life cycle and phenology of an Antarctic invader: the flightless chironomid midge, *Eretmoptera murphyi*

**DOI:** 10.1007/s00300-018-2403-5

**Published:** 2018-09-29

**Authors:** Jesamine C. Bartlett, Peter Convey, Scott A. L. Hayward

**Affiliations:** 1 School of Biosciences, University of Birmingham, Edgbaston, B15 2TT UK; 2 British Antarctic Survey, NERC, High Cross, Madingley Road, Cambridge, CB3 0ET UK

**Keywords:** Chironomidae, Signy Island, Embryogenesis, Pupal development, Population growth

## Abstract

Knowledge of the life cycles of non-native species in Antarctica is key to understanding their ability to establish and spread to new regions. Through laboratory studies and field observations on Signy Island (South Orkney Islands, maritime Antarctic), we detail the life stages and phenology of *Eretmoptera murphyi* (Schaeffer 1914), a brachypterous chironomid midge introduced to Signy in the 1960s from sub-Antarctic South Georgia where it is endemic. We confirm that the species is parthenogenetic and suggest that this enables *E. murphyi* to have an adult emergence period that extends across the entire maritime Antarctic summer season, unlike its sexually reproducing sister species *Belgica antarctica* which is itself endemic to the Antarctic Peninsula and South Shetland Islands. We report details of previously undescribed life stages, including verification of four larval instars, pupal development, egg gestation and development, reproductive viability and discuss potential environmental cues for transitioning between these developmental stages. Whilst reproductive success is limited to an extent by high mortality at eclosion, failure to oviposit and low egg-hatching rate, the population is still able to potentially double in size with every life cycle.

**Electronic supplementary material:**

The online version of this article (doi:10.1007/s00300-018-2403-5) contains supplementary material, which is available to authorized users.

## Introduction

The sub-Antarctic islands, with a longer history and greater level of human influence than any other part of the Antarctic (Convey 2013), have a greater number of non-native species than the more extreme maritime and continental Antarctic regions further south (Convey and Lebouvier 2009; Frenot et al. 2005). However, in recent years and decades, there have been increasing records of species establishing in the maritime Antarctic with anthropogenic assistance, particularly in the South Shetland Islands and northern Antarctic Peninsula (e.g. Greenslade et al. 2012; Volonterio et al. 2013; Hughes et al. 2015; Molina-Montenegro et al. 2012). With synergy between high and increasing levels of human activity in this region of the Antarctic, and recent rapid rates of regional climate change, further establishment of non-native species is predicted, presenting fundamental challenges to the protection and conservation of Antarctic terrestrial biodiversity, and to the management and governance processes in the Antarctic (Chown et al. 2012; Chown and Convey 2016; Hughes and Worland 2010; Tin et al. 2009).

The brachypterous midge *Eretmoptera murphyi* (Chironomidae, Orthocladiinae) is a non-native species on Signy Island (South Orkney Islands, maritime Antarctic), to which it is thought to have been inadvertently introduced in the 1960s, in association with plant transplant experiments (Block et al. 1984; Convey and Block 1996). Its larvae have the capacity to rapidly cold harden, cryoprotectively dehydrate (Everatt et al. 2012, 2015; Worland 2010), respire in water and withstand ice entrapment (Everatt et al. 2014). These traits have allowed it to succeed in the maritime Antarctic, which is more extreme in comparison with the species’ native sub-Antarctic South Georgia. The sub-Antarctic has a relatively stable and chronically cool oceanic-influenced climate year-round. This presents fundamentally different pressures for terrestrial invertebrates to that of the much more extreme seasonality of the maritime Antarctic, where overwintering microhabitat temperatures can regularly fall below −10 °C, contrasting with minima only marginally below zero on South Georgia (Convey 1996a; Convey and Block 1996). To date, studies of *E. murphyi* have primarily focussed on the ecophysiology of late instar larvae (Everatt et al. 2012, 2015; Worland 2010; Hughes et al. 2013). However, a much more detailed characterisation of all life stages is required to determine how current and predicted future climate changes may affect this species’ development and phenology.

### Life history strategies of polar arthropods

Driven by the short growing seasons and environmental extremes, polar invertebrates often exhibit ‘adversity-selected’ life history strategies in comparison with their temperate counterparts (Convey 1996b). They have slow growth rates (Convey 1996b), extended and free-running life cycles with reduction of obligate overwintering stages (Fogg et al. 2008), considerable investment in stress tolerance mechanisms (Convey 1996b; Hayward et al. 2003; Everatt et al. 2015) and the ability to opportunistically take advantage of even short periods of conditions suitable for growth and activity; for instance, the Antarctic oribatid mite, *Alaskozetes antarcticus*, has a life cycle duration of around 5 years, whilst comparable temperate species are typically annual or biennial (Convey 1994; Block and Convey 1995). Consequently, multi-year life cycles are common in polar arthropods and many lack a true diapause, instead entering a state of temporary quiescence during winter or other shorter periods of unsuitable conditions. Thus, the most commonly shared life history feature across polar arthropods is the flexibility which enables the challenges of adverse conditions to be overcome, although some ‘programmed’ elements may remain so that key life stages can take advantage of regular environmental triggers each season (Convey 1996a, b; Danks 1999; Worland and Convey 2008).

Chironomid midges are a group of higher insects that are particularly well represented at high latitudes in both hemispheres relative to other insect groups (Chown and Convey 2016; Convey and Block 1996; Coulson et al. 2014). Polar representatives typically conform to the normative polar life history strategy as defined by Danks (1999), having a fixed and synchronous spring emergence after overwintering in a late larval stage, and a brief adult reproductive stage during summer, but an otherwise flexible life history. Asexual reproduction is prevalent in all major polar arthropod and microinvertebrate groups (Chown and Convey 2016; Convey 1996a) and especially so in sub-Antarctic Psychodidae, a family of biting midges (Duckhouse 1985). However, asexual reproduction has not yet been definitively proved in any maritime Antarctic insect species (Convey 1996a) despite being strongly suspected in *E. murphyi* (Convey 1992; Cranston 1985).

### Life histories of Antarctic chironomids

The life histories and biology of the native Antarctic chironomids Parochlus steinenii (Gercke 1889) (Podonominae) and *Belgica antarctica* (Jacobs 1900) (Orthocladiinae) have been well studied (e.g. Allegrucci et al. Allegruci et al., 2006, 2012; Convey and Block 1996; Harada et al. 2014; Hahn and Reinhardt 2006; Sugg et al. 1983; Usher and Edwards 1984). These are typically characterised by larval development taking place over 2 years, overwintering as either early or late instars, followed by synchronised mass emergence of adults in summer (Convey and Block 1996; Harada et al. 2014; Sugg et al. 1983). *Belgica antarctica* occurs along the Antarctic Peninsula and is the only higher insect endemic to the Antarctic continent (Convey and Block 1996; Kelley et al. 2014). It experiences environmental conditions similar to those of *E. murphyi* on Signy Island, and the assumption is that both species have similar ecological niches. Recent molecular evidence also suggests that *E. murphyi* should be assigned to the genus *Belgica* (Allegrucci et al. 2012), further supporting likely common life history strategies. However, questions remain as to whether the long evolutionary history of *E. murphyi* on sub-Antarctic South Georgia has provided the opportunity for the evolution of a temperate-style life history pattern that would show less flexibility than that of a more typical polar insect.

In the field on Signy Island *E. murphyi* is thought to emerge en masse, possibly in response to abiotic factors such as increased spring daylength, the seasonal melt of basal snow (Block et al. 1984; Gardiner et al. 1998) or as a heritage trait from related chironomids (Armitage et al. 1995). Convey (1992) showed that rates of egg development decrease with an increase in temperature (2–12 °C) and that the females invest greatly in reproduction with ca. 85 eggs being laid in a single hydrosensitive egg sac—representing a dry mass twice that of the female post-oviposition. Once larvae hatch they are thought to overwinter twice (Hughes et al. 2013; Worland 2010), once in an early larval stage and later in the fourth instar, although this has not been explicitly demonstrated. It is assumed that *E. murphyi* has four larval instars like *B. antarctica*, although previous size class distribution analyses and taxonomic studies have identified only two distinct classes via assessments of larval mass or field observations (Cranston 1985; Hughes et al. 2013). One reason underlying the current lack of explicit knowledge of *E. murphyi*’s life history has been the challenge of establishing a long-term laboratory culture, with all data obtained to date derived from short periods of field observations combined with laboratory experiments relying on field-collected material (Convey 1992; Everatt et al. 2014; Hughes et al. 2013).

### Patterns of climate change in the western Antarctic Peninsula and Scotia Arc

In recent decades, rapid regional warming and other physical environmental changes have been documented in parts of Antarctica, in particular in the region of the western Antarctic Peninsula and Scotia Arc (Turner et al. 2009, 2014), including Signy Island (Cannone et al. 2016; Royles et al. 2012; Smith 1990). Signy Island was recognised early on as a paradigmatic location at which to study terrestrial biological processes in the maritime Antarctic, and how these might change under the influence of changing environmental drivers (Smith 1990). Within terrestrial ecosystems, the primary consequences of these environmental changes are longer active seasons (earlier spring thaw combined with later autumn freeze), greater integrated thermal energy availability (increased temperatures) and greater availability of liquid water to terrestrial organisms. Thus, and unlike the general consequences in many regions of the world, regional warming in parts of the Antarctic relaxes the current extreme environmental constraints on biological processes, and recent syntheses recognise that many of the native biota in these regions, including polar terrestrial invertebrates, are likely to benefit from the changes being observed (Bale and Hayward 2010; Convey 2011; Convey et al. 2014). It is also increasingly recognised that this relaxation of environmental constraints, with or without the direct influence of human assistance in transporting propagules, will lower the barriers to new species arriving and establishing in Antarctica (Frenot et al. 2005; Hughes et al. 2006).

### Aims of this study

Against this background, the primary aims of this study are to provide the first detailed characterisation of different developmental stages within the life cycle of *E. murphyi*, and to investigate the potential role of abiotic triggers in the timing of major life history transitions on Signy Island, such as pupation, adult eclosion or oviposition. These are then considered in the context of the implications of climate change for this species’ life history and distribution on the island and, potentially, more widely in the maritime Antarctic.

## Materials and methods

### Sample collection and processing

**Fig. 1:**
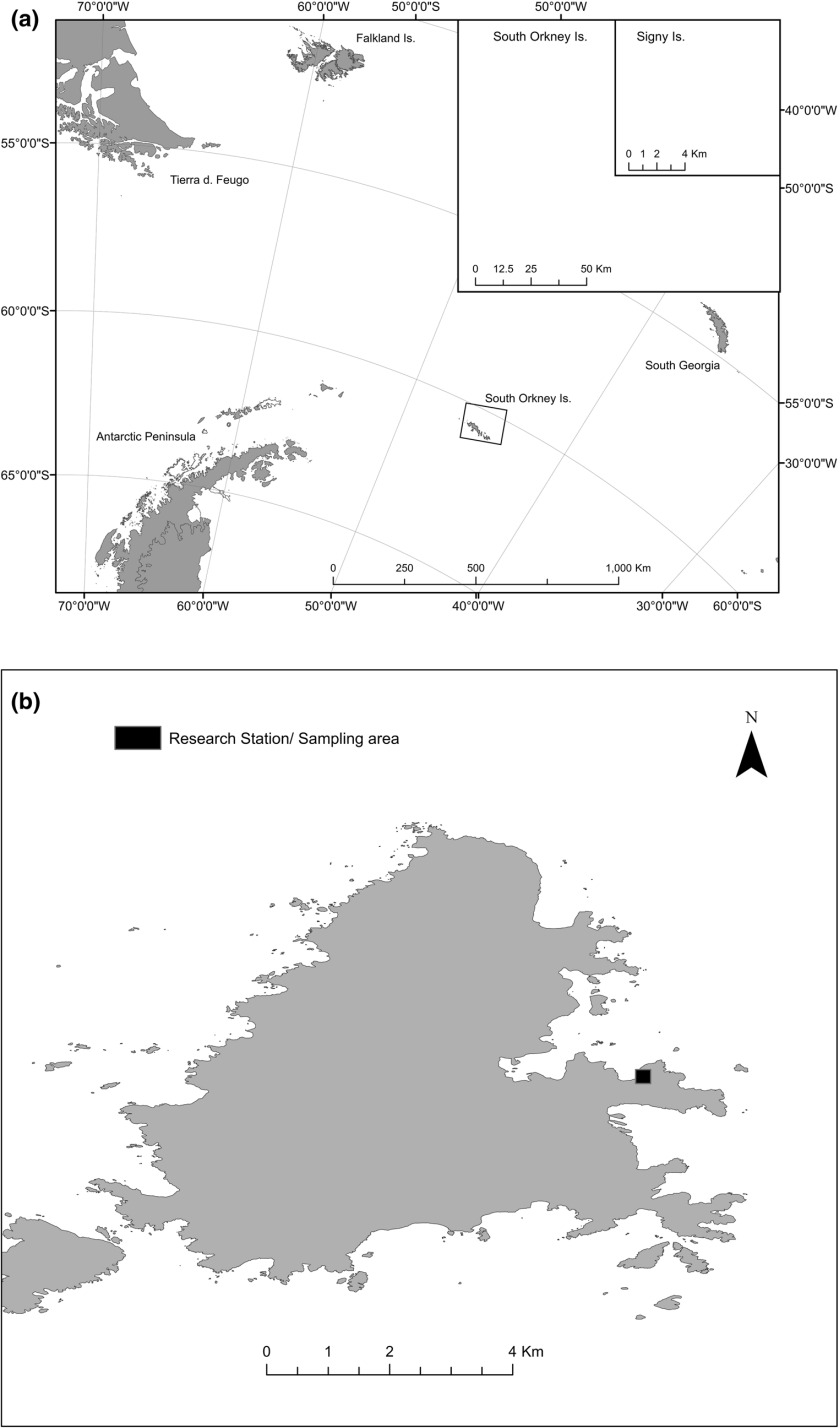
Maps showing the Location of the South Orkney Islands and Signy Island in the Southern Ocean. Inset—Map of Signy Island, with Research Station (and thus current area of *E. murphyi* distribution) highlighted. Created using ArcMap®10.4.1 software by Esri. Copyright © Esri

All samples were either obtained from, or observed in situ, on the Backslope and in the immediate vicinity of the British Antarctic Survey (BAS) research station on Signy Island (South Orkney Islands, maritime Antarctic, 60°43′0″S, 45°36′0″W; Fig. 1a, b). Samples collected during the 2014/2015 austral summer by BAS staff were returned to the United Kingdom by ship in + 4 °C cold storage (10 weeks), and then maintained at + 4 °C at the University of Birmingham until use. Studies were conducted in the field on Signy Island between December 2016 and March 2017. All laboratory cultures and experiments, both at the University of Birmingham and on Signy Island, were maintained on local Signy peat soil substrate, which is both the species’ habitat and food source on the island. The substrate was kept moist with a soil solution comprising 3:1 deionised water to Signy soil (hereafter termed ‘field water’) to ensure that conditions deviated as little as possible from the natural environment.

Eretmoptera murphyi’s current distribution on Signy Island is centered around the research station and adjacent Backslope, and therefore all monitoring and sampling occurred within a few hundred metres of the station (Fig. 1b). All images and morphological measurements were obtained using a Leica EZ4 digital microscope and associated software. Individual larvae or adults were extracted from the soil/moss substrate by washing through stacked sieves (2-mm, 0.5-mm mesh sizes) and handpicked from the remaining soil solution. Moss and peat substrate was broken apart with fine tweezers prior to washing to ensure individuals were not trapped amongst the fibres. Weather conditions were noted in association with all field experiments and collection days, with particular attention to recording strong sunshine and significant precipitation events.

### Measurement of larvae

Larvae were assigned to instars based on size. They were initially separated into approximate size classes by eye, followed by detailed width and length measurements using images taken with a digital microscope with in-built camera (Leica EZ4). The microscope software was calibrated for each image using a micrometre stage graticule. Width measurements were taken by measuring the length of the intersegmental groove between segment IV (SIV) and segment V (SV)—the intersection of the cephalothorax and abdomen. Length measurements were taken from head to anus, but did not include mandibles or posterior parapods (the latter only in the case of L1). This information on distinct size classes then informed the selection of L4 larvae for studies of pupal development and larval instar occurrence in phenological surveys, described below.

### Environmental triggers for pupation

In laboratory samples maintained at 4 °C under constant darkness, progression to pupae from the L4 instar is infrequent and unpredictable (PC, JB, pers. obs.). We therefore hypothesised that other environmental signals might be required to trigger pupation. In a simple test of this, batches of L4 larvae (*n* = 20) were placed under the following conditions representing different temperature, light and water availability scenarios on Signy Island during the transitions from winter to spring and summer (Table 1). Control conditions were constant darkness at 5 °C. To determine if light was a trigger for pupation, samples were transferred to 19L:5D (5 °C), which approximates summer photoperiods on Signy. A fluctuating temperature regime of 5 °C during illumination and 0 °C during darkness was also used, to approximate typical Signy summer diurnal conditions. To determine if spring melt/access to water was a trigger for pupation, larvae were maintained either under “wet” conditions (1:1 soil mass-to-‘field water’ volume ratio) or “dry” conditions (no additional water was added to the substrate) in petri dishes.

**Table 1:** Environmental treatments used to assess influence of temperature, light and soil saturation on pupation

Dominant light condition	Temperature (°C )	Light regime (L:D)	Soil moisture
Light	5	19:5	Wet
			Dry
Light	2	19:5	Wet
			Dry
Dark	5	0:24	Wet
			Dry
Dark	5–0	19:5	Wet
			Dry

Larvae were maintained under these experimental conditions for 60 days from 18 December 2015 to 18 February 2016, and cumulative pupation recorded. Any pupae obtained were maintained under the same temperature and light conditions, but with saturated soil, until either death or eclosion to imago.

### Pupal development

Initial observations suggested morphologically distinct phases of pupal development, so *n* = 31 individual pupae were observed and imaged as they occurred in laboratory stocks throughout the study (i.e. from both the 2014/2015 BAS collection and 2016/2017 collections). To clearly document the discreet phases, digital images were taken of all pupae under the different treatments applied, with width and length data recorded as well as other key morphological and physiological changes including development of gonads, development from stemmata to compound eyes and changes to cuticle pigmentation (Table 2). This definition of pupal phases informed the experimental design for field monitoring of pupal and imago development during the 2016/2017 season.

**Table 2:** Description of pupal phases and classification guide for development tracking

Pupal Phase	Size (mm)	Physically mobile	Eye type	Pigmentation	Legs	Gonapophysis	Reproductively viable	Development time in phase (days)
1	1–1.5	Very	S	None	Sheathed	None	No	3.00 ± 0.82
2	1.5	Somewhat	S&C	Cephalothorax & legs	Sheathed	Yes	No	4.67 ± 2.75
3	2	Sessile	C	Full, opaque	Sheathed	Yes	No	2.14 ± 2.59
4	> 2	Somewhat	C	Full, opaque	Free	Yes	Yes	1.00 ± 1.31

Average development time within each phase ± SEM. *n* = 31 pupae assessed in laboratory conditions (5 °C, saturated soil, dark). Eye type: S = stemmata; C = compound eye. Size is total body length

### Pupal and imago development in the field

Field monitoring of pupal development took place during January 2017 adjacent to Signy Research Station. Individual pupae (*n* = 20) were placed in open petri dishes containing local substrate within a larger arena placed on the ground, and temperature data were recorded for the duration of the observations. The arena was constructed using 2-L plastic tubs with modified lids of nylon mesh, in order to keep the arena open to the environment whilst preventing damage by local wildlife, predominantly the Brown Skua (*Stercorarius antarcticus*). Pupae were assessed daily from 20 December 2016 to 6 January 2017. Temperature readings inside the arena were taken each day at the time of surveying using a soil temperature probe and digital thermometer (RS Pro-206-3738 with Type K thermocouple probe) and ambient readings collected with an adjacent temperature logger external to the arena (Tinytag Transit TG-0050). Pupae were followed through their development via assessment with a hand lens and allocated to the developmental stages as noted above and in Table 2, followed by recording their eclosion date and any subsequent oviposition by adults.

### Adult emergence

Three 0.5-m^2^ quadrats were set out on a moss bank adjacent to the research station and all were monitored twice daily for 5 min at 1000 and 1600 local time, noting the presence of adults active on the surface, between 20 December 2016 and 6 January 2017. Ground surface temperature readings were taken each day during the observation periods, as described above.

### Egg maintenance and larval development

Laboratory cultures established from the 2014/2015 stocks enabled the rearing of larvae to pupation and subsequent emergence with successful oviposition. From these laboratory eggs, an initial four-phase classification system of embryonic development was established to aid development stage identification (Table 3). All laboratory adults that emerged and then oviposited under one of the experimental environmental conditions described above were subsequently maintained with their egg sacs at 5 °C on saturated substrate to maintain sac structure. Hydrated egg sac diameters were measured, and numbers of eggs were recorded. Eggs were assessed every 48 h, and developmental stage recorded.

**Table 3:** Description of egg development stages and classification guide for development tracking

Egg stage	Image	Description	Development (days)	Success rate (%)
1—Opal		Opaque white eggs granulated and slightly iridescent in appearance. No pigmentation	10–14	66 ± 2.72
2—Yellow		Outer-casing turning yellow/brown. Still granulated and no sign yet of embryonic form.	5–7	100
3—Early embryo		Shape of embryo becomes clearer and red stemmata eyes become evident.	7–10	92.2 ± 0.97
4—Late embryo		Pharate larva visible with some evidence of internal organs, eyes and mandibles clearly visible.	7–10	82 ± 2.29

Typical duration of each stage given and mean success rate/progression to next phase ± SEM. Success rate = % that successfully complete each development stage

### Monitoring of egg development in the field

Recently laid egg sacs (*n* = 11) collected from field samples were placed in open 2-cm petri dishes with saturated substrate in an external arena and monitored every 48 h until development ceased, or the eggs hatched (a maximum of 39 days), and then again on day 45 to confirm that no further delayed development had occurred. Temperature readings inside the arena were noted daily as above. The development stage of each egg (Table 3) within each egg sac was recorded using a dissecting microscope. On day 45, all egg sacs were dissected, and any remaining unhatched eggs individually inspected. A total of *n* = 740 eggs were assessed.

### Phenology of summer-occurring life stages

Weekly soil cores (*n* = 5) were taken from a site adjacent to the research station where *E. murphyi* was abundant, using a steel 5-cm × 10-cm corer. This took place between 23 January and 6 March 2017 (= late summer season). Soil cores were returned to the Signy laboratory in a sterile sealed bag and processed within 24 h. Cores were divided into vegetation and soil/peat substratum and weighed using a Sartorius precision balance (E6202) before being carefully washed separately through stacked sieves as described above. All life stages were extracted, sorted into groups (adults; pupae; L4 larvae; L3 larvae; L2 larvae; eggs unhatched, eggs hatched) and counted. L1 larvae were not included as their small size would have resulted in sample processing being too time consuming. All sieved substrate was dried for 24 h at 60 °C and re-weighed to obtain constant dry mass, against which all counts were normalised. An additional core was collected each week, divided into vegetation and soil components and used to make pH and salinity measurements with a Hanna combo water reader (HI-98129). Throughout the field period in 2016/2017, the presence of pupae or adults on the surface were recorded, from which the final dates of sighting of both pupae and adults were established.

## Results

### Environmental description

The Signy field site was generally very stable throughout the 2016/2017 season. The mean pH in the vegetation layer was 5.3 ± 0.13 SE, *n* = 7, and underlying soil pH was 5.5 ± 0.11 SE, *n* = 7. Salinity was also largely stable, with only one spike during a week of high storm activity detected in the vegetation layer, when it rose to 425 µS from an average of 174 ± 45 µS SE, *n* = 7. Salinity in the soil layer remained close to 70 ± 10 µS SE, *n* = 7.

### Larval classification

**Fig. 2:**
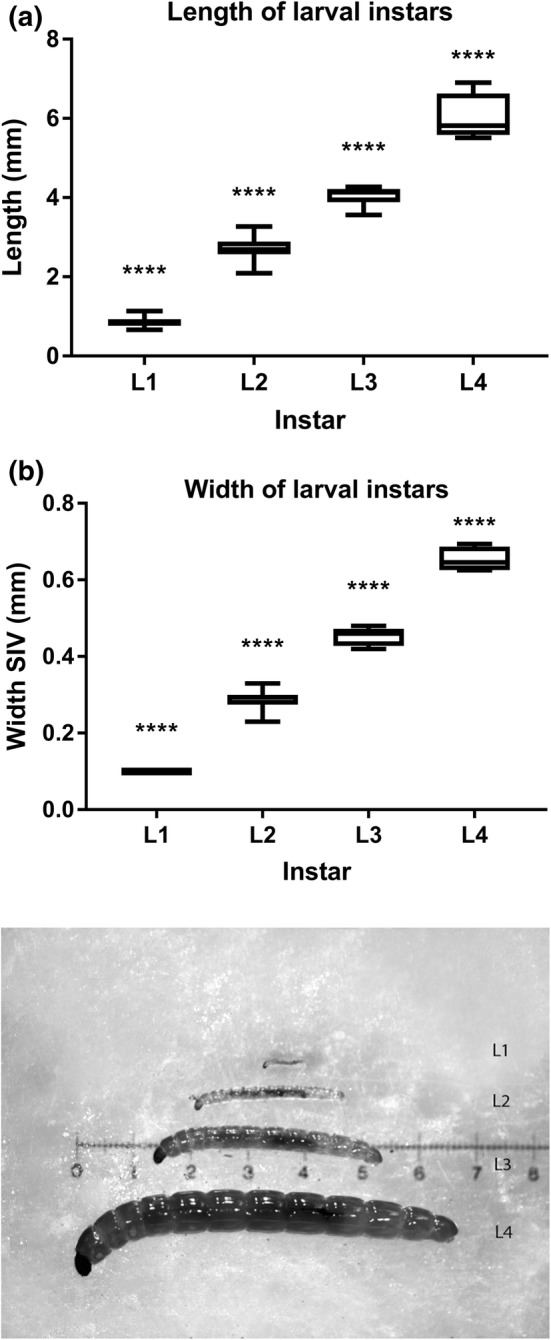
Classification of the four larval instars by **a** total body length and **b** width at segment four/five intersection; *n* = 10 individuals for each of L1, 3 and 4, *n* = 12 individuals for L2. All instars significantly different from each other for length: ANOVA *F*(3,31) = 372.2, *p * < 0.0001; width: ANOVA *F*(3,34) = 780.7, *p* < 0.0001. **c** Larval instars side by side: Top to bottom, L1 to L4

Size class analysis proved to be suitable for separating the four larval instars, with each size class being significantly different from each other in both width at SIV and length (Fig. 2), and no overlap between instar size classes.

### Larval survival and environmental triggers for pupation

**Fig. 3:**
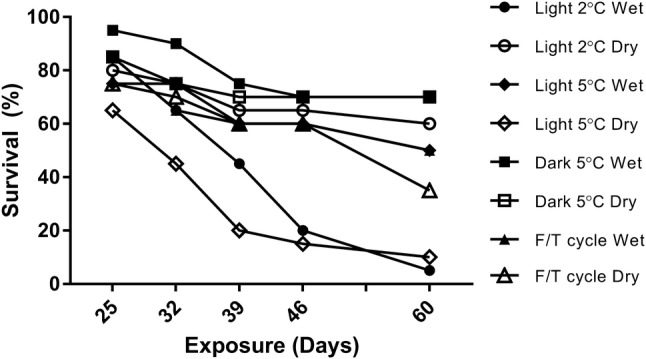
**a** Larvae survival over 60 days after exposure to varied light, temperature and substrate saturation levels (*n* = 20 for each condition). Survival over time across all treatments (2-way ANOVA Tukey’s post hoc comparisons, *F*(4,20) = 11, *p* = < 0.0001). Soil moisture effect the only significant variable in Light 5 °C (Mann–Whitney *U*, *p* = 0.046). **b** Total pupations from larvae during experimental conditions (Kruskal–Wallis, H = 13.7; *p* = 0.6)

Larval survival (Fig. 3) was greatest in the control/dark 5 °C conditions (70% survival after 60 days), and was significantly different from the light treatments (two-way ANOVA with Tukey’s multiple comparisons column factor, *F*(3,20) = 9.71: Light 2 °C—32.5% survival after 60 days, *p* = 0.008; Light 5 °C—30% survival after 60 days, *p* < 0.001). Overall survival dropped significantly over time across all treatments (two-way ANOVA with Tukey’s multiple comparisons, *F*(4,20) = 11, *p* < 0.0001) and soil moisture only had a small effect on survival in the warmest lit conditions of Light 5 °C (Mann–Whitney *U* = 3, *p* = 0.046,). Of all environmental conditions tested, the fluctuating freeze/thaw cycle of + 5 °C/0 °C with corresponding 19:5/L:D, which is the condition most reflective of Signy summer conditions, led to the greatest level of pupation, although this was not significantly different from other treatments (Kruskal–Wallis, *H* = 13.7, *p* = 0.6).

### Pupal classification and development

Pupae exhibited four distinct phases of development before completing the molt to imago (Table 2). Broadly, the first and second phases were differentiated by an increased level of pigmentation and development of the gonads. The third phase was quite sessile, deeply pigmented and with the legs still encased in leg sheaths. There was a thickening of the cuticle in this stage. The final fourth phase was a partial eclosion, where the legs were free of the sheath but the imago not fully eclosed from the exuvia. Mean development time in the field from initial pupation to eclosion was 14 days (± 5 days, *n* = 12), with the longest period spent in the seconnd phase of pupation (Table 2). There was no difference in development rates of pupae incubated at constant or fluctuating temperature in laboratories in the UK, compared with those in the field conditions with a fluctuating temperature on Signy Island (Kruskal–Wallis, *H* = 1.3, *p* = 0.54).

### Eclosion, imago development and phenology

Only 45% of pupae (*n* = 20) placed within the external field arenas successfully eclosed, and 55% of these adults oviposited. There was no correlation between numbers of individuals eclosing and either ambient temperature on the ground surface over the preceding 24 h or within the pupation arena at the time of sampling (surface temperature: *r*_s_ =0.21, p = 0.4; arena temperature: *r*_s_ =0.31, *p* = 0.2). There was, however, a strong correlation between the temperature outside the arena and the spot temperature taken within it at the time of surveying, verifying that the arena did not increase temperature artificially (*r*_s_ =0.88, *p* < 0.0001). Monitoring of quadrats for the presence of adults showed no correlation with daily ambient mean temperatures (*r*^2^ = 0.07), although anecdotally adult presence was associated with calm clear days (Online Resource 1).

### Egg classification and monitoring

**Fig. 4:**
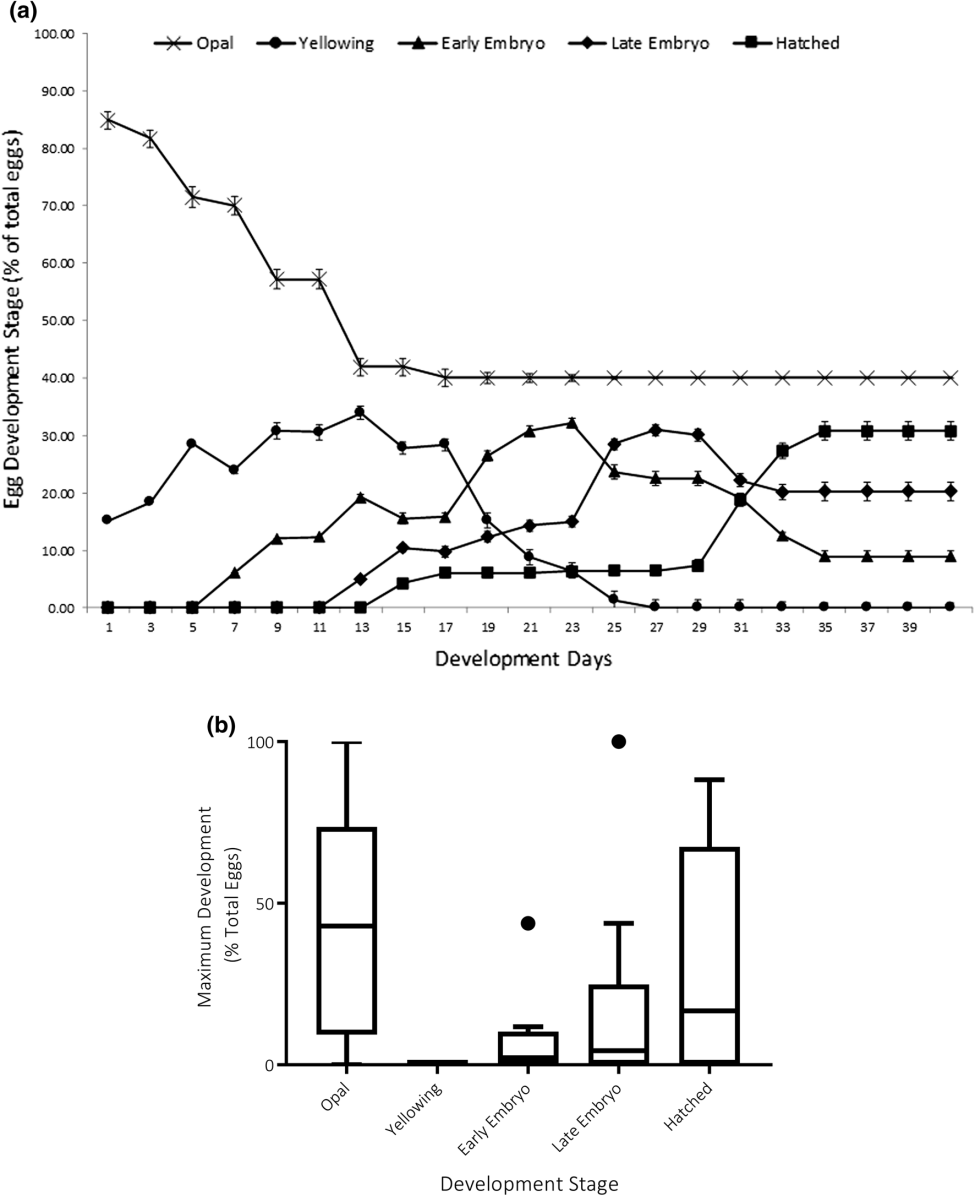
**a** Egg development in field conditions on Signy Island. Stages of individual egg (*n* = 740) development within each egg sac (*n* = 11) recorded over time, shown with CI of 95%. **b** Maximum stage reached after 45 days of monitoring (Tukey’s plot) with outliers

**Fig. 5:**
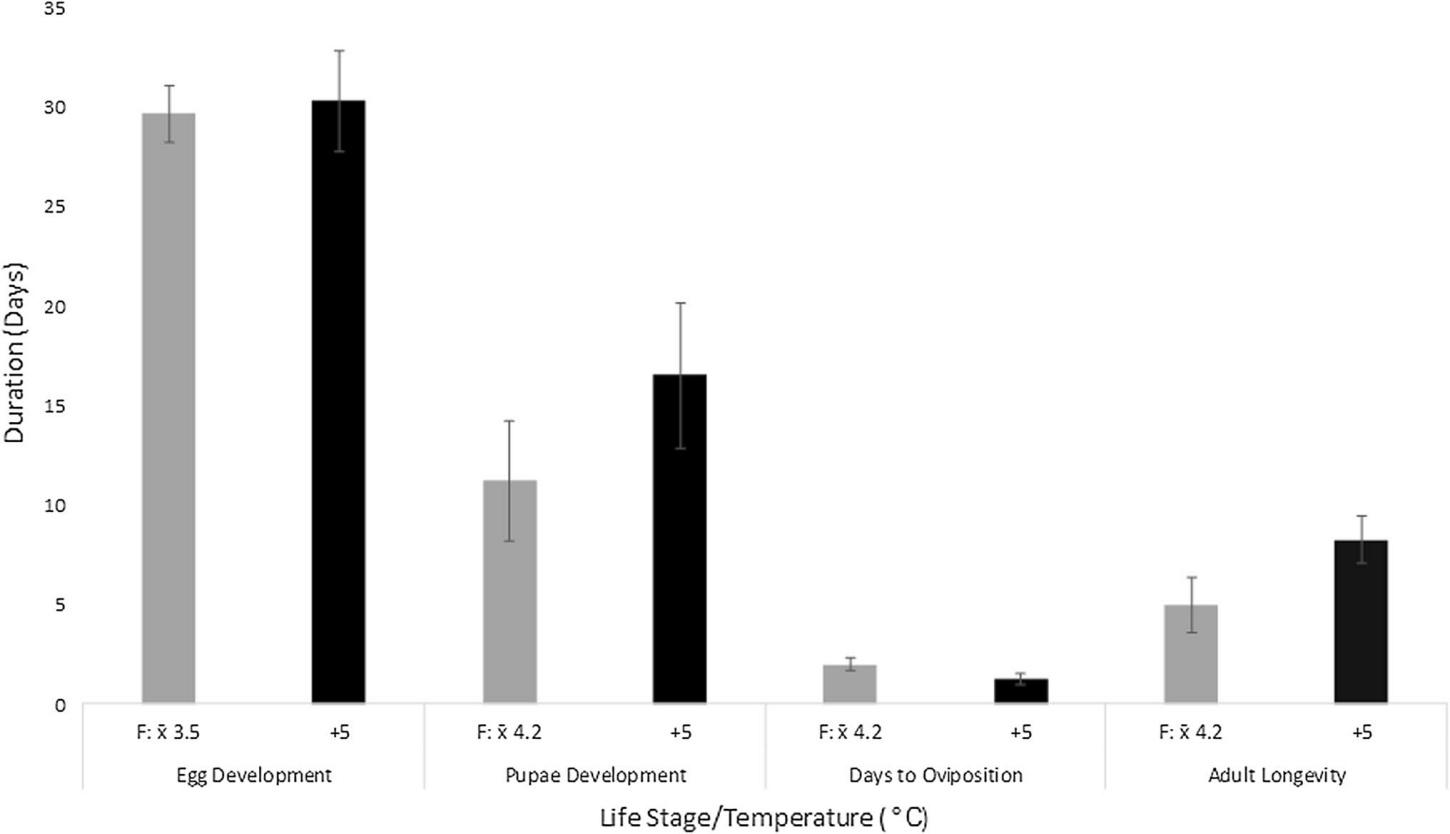
Mean (± SE) time (days) taken to complete egg or pupal stage under different temperature conditions. Also shown, the mean period from eclosion to oviposition and adult longevity post eclosion. Field conditions are shown as the mean temperature experienced (*F*: *x̄*) over the relevant period. Pupae and adult development periods overlapped and thus were subject to the same average field temperatures. Lab temperatures were either static 5 °C or 12h 5/12 h 0 °C. Sample sizes—Egg development: “*F*: *x̄* 3.5″, *n* = 6 egg sacs; “+ 5″ *n* = 7. Pupae development: “*F*: *x̄* 4.2″ *n* = 5; “+ 5″ *n* = 4. Oviposition “*F*: *x̄* 4.2″ *n* = 5; “+ 5″ *n* = 4. *Adults* “*F*: *x̄* 4.2″ *n* = 5; + 5 *n* = 4

Egg sacs (*n* = 30) had a mean dry mass of 0.14 mg (± 0.06 mg) and water content of 96% (± 1.29%) fresh mass. Egg sacs contained a mean of 48 (± 12.48 *n* = 30) individual eggs and had a diameter of 1.78 (± 0.4 *n* = 30) mm, with size not being significantly correlated to either number of eggs or water content (*r*^2^ = 0.08 and 0.009, respectively). Changes in the proportion of different egg stages within egg sacs (Table 3) until hatching are presented in Fig. 4a. Stage 1 (opal) spanned c. 14 days across all samples, although very consistently across all egg sacs approximately 40% of eggs did not develop beyond this stage (Fig. 4b). The eggs then turned yellow, before the early embryo with stemmata evident become visible around day 19. Late embryos, with visible mandibles and pharate larvae, appeared around day 25 and eggs hatched by day 31. Development within an individual egg sac was not tightly synchronised. After 45 days of field observations of *n* = 740 eggs, 40% (± 2.72%) did not progress past the first ‘opal’ stage (Fig. 4b). Nearly all remaining eggs progressed through the yellowing phase, but 7.6% (± 0.97%) did not progress beyond the early embryo and 16% (± 2.29%) beyond the late embryo, with only 35% of all eggs (± 2.44%) going on to hatch. There was no difference in development rates of eggs incubated at constant temperature compared with those under field conditions with fluctuating temperature (Mann–Whitney *U* = 17, *p* = 0.62) (Fig. 5).

### Phenology and environmental factors

**Fig. 6:**
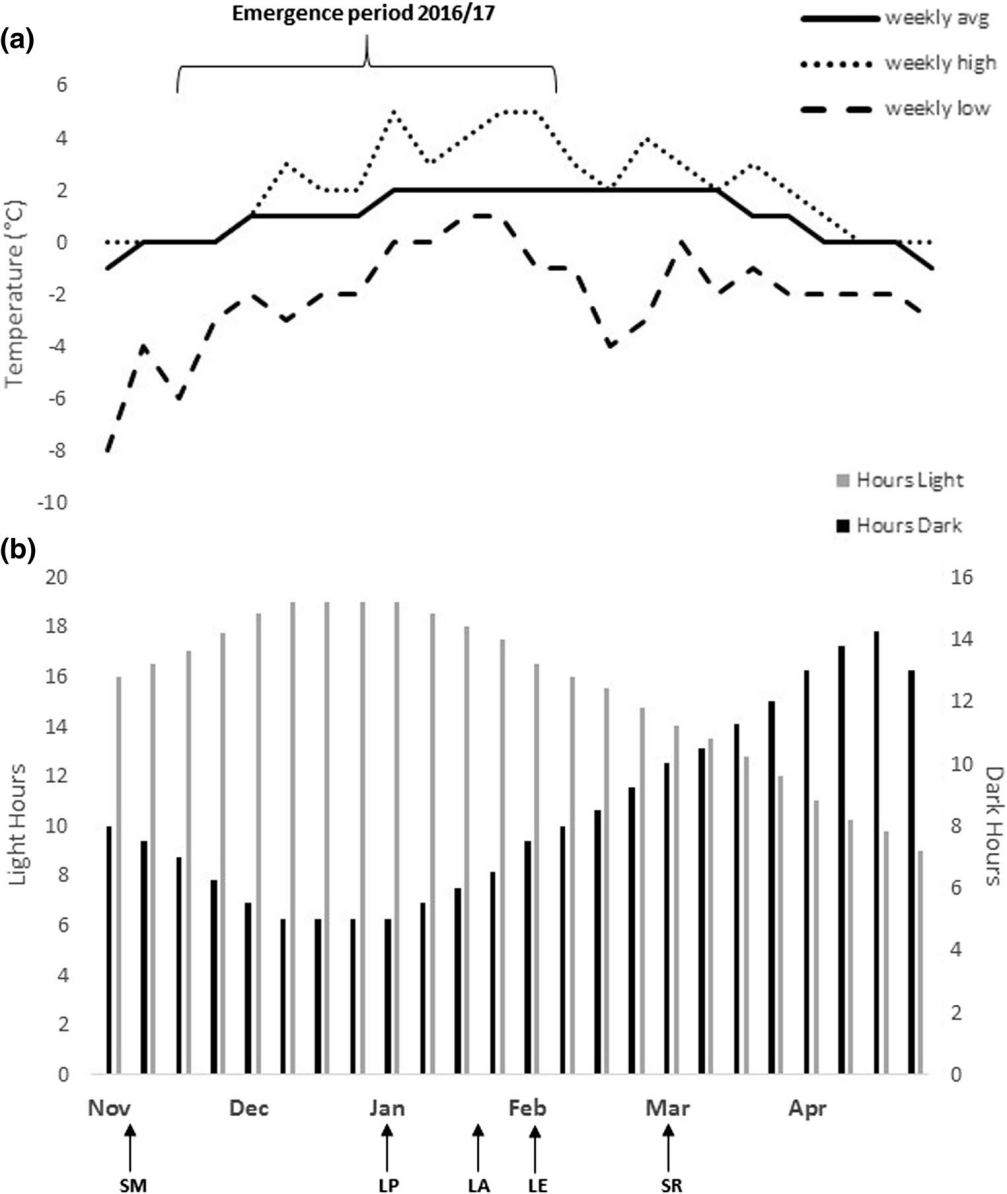
**a** Mean (weekly) hours of daylight and darkness as well as high and low air temperatures on Signy Island, annotated with key points in the development of *E. murphyi* life stages: *SM* basal snow melt, mid Nov 2016; *LP* last pupae seen 3 Jan 2017; *LA* last adult seen 19 Jan 2017; *LE* last unhatched eggs seen 30 Jan 2017; *SR* snow returned 27 Feb 2017. **b** Abundance of different *E. murphyi* life stages found in soil cores from a single site collected from late January to the beginning of March 2017, shown as mean percentage of total population (±SE). *A* adults, *P* pupae; *L2, L3 and L4* larval instars; *EU* egg sacs where majority (> 50%) of eggs were unhatched, *EH* egg sacs where the majority (> 50%) of eggs had hatched

Seasonal life-stage monitoring in the field over the late summer showed that the *E. murphyi* population had progressed through pupal and adult stages before the end of January (Fig. 6a). The last pupae were found on 3 January 2017 and the last adult was seen on 19 January 2017 (Fig. 6b). By 13 February 2017, no further viable unhatched eggs were found in the samples (determined by the presence of hatched eggs alongside those that had stalled development at a much earlier stage), with only hatched eggs collected thereafter. Unhatched eggs that were considered undeveloped/unviable appeared to decompose, becoming opaque, soft and bloated, often combined with visible fungal development. The phenological soil core surveys did not include any counts of adults or pupae (Fig. 6b), so these were discounted from analysis. There was no significant difference in the appearance of the larval instars or trend over time (Kruskal–Wallis, *H* = 1.3, *p* = 0.53; *r*^2^ = 0.1). The appearance of unhatched and then hatched eggs did show a visible difference (Fig. 6b), with a distinct increase in the number of hatched eggs over time compared to a decline in unhatched (Mann–Whitney *U* = 202, *p* = 0.008).

### Population success

Analysis of reproductive output (Table 4) suggests that the *E. murphyi* population can potentially double with each life cycle. Considering the percentage of larvae that survive over 60 days, pupae that successfully eclose, adults that successfully oviposit and the viability of eggs laid, this will amount to an average 50,000 additional L1 added to the population every two years, based on the most recent distribution data reported by Hughes and Worland (2010) (Table 4).

**Table 4:** Life stage success table using population densities of larvae reported by Hughes and Worland (2010)

Population density	Larvae density (m^2^)	Approximate larvae survival (m^2^)	Successfully eclose (m^2^)	Successfully oviposit (m^2^)	Total Eggs laid (m^2^)	Total Hatched (m^2^)
High	150,000	105,000	47,250	25,987	1,247,400	361,746
Average	21,000	14,700	6,615	3,638	174,636	50,644
Low	500	350	157	87	4,158	1,205

Average larval densities are taken from the whole sample site. ~70% of L4 larvae survive; 45% of larvae successfully eclose, 55% then oviposit, with a mean of 48 eggs per oviposition and 35% go on to hatch

## Discussion

### Larval and pupal development

The developmental stages described here for larval instars are consistent with the only taxonomic study of *E. murphyi* (Cranston 1985) and provide a first description of the L1 and L2 instars. Like the sister species *B. antarctica* (Sugg et al. 1983), and typical for chironomids, there are four larval instars, which in our data do not overlap in size classes for either width or length and provide a clearer assessment of all instars than the use of body mass classes (cf. Hughes et al. 2013). Our observations of pupae indicate that metamorphosis takes around 14 d, and can be divided into four morphologically distinct phases, providing a new level of detail for *E. murphyi* and for chironomids in general, whose pupal stage is understudied (Armitage et al. 1995). Apparent obligate parthenogenesis in *E. murphyi* enables oviposition to occur prior to the completion of eclosion. Whilst this alone is not unique among asexual chironomids (Armitage et al. 1995; Langton et al. 1988), it does offer *E. murphyi* a distinct advantage in a polar habitat such as on Signy Island, enabling it to reproduce even if conditions or physiology do not permit eclosion. Our data do not provide evidence for a temperature or moisture/spring thaw trigger for pupation. Fluctuating temperatures of the spring season (+ 5/0 °C), did result in more pupae; however, this was not statistically different from other treatments. Block et al. (1984) reported that pupae of *E. murphyi* appeared shortly after spring thaws, and a similar observation has been reported in an aquatic Antarctic midge, *Parochlus steinenii* (Hahn and Reinhardt 2006; Rauschert 1985), but further early season sampling will be required to clarify the cues required to initiate pupation.

### Adult emergence and parthenogenesis

The emergence of *E. murphyi* adults does not take place in a synchronous mass event as reported in *B. antarctica* (Sugg et al. 1983) but continues over a 2–3-month period (Online Resource 1). In the 2016/2017 summer season, adults were already noted to be active when Signy station was opened in mid-November (Station Leader M. Jobson, pers. comm.), coinciding with an early spring thaw. Hatched eggs were already present in the soil in late December 2016 which, based on the egg development times recorded in this study, means they would have been laid in late November at the earliest. This could give *E. murphyi* a distinct advantage over sexual reproducers, such as *B. antarctica*, the only chironomid that successfully completes its life cycle on the Antarctic Peninsula. Whilst *B. antarctica* is limited by the need to have males and females emerge synchronously to reproduce sexually, *E. murphyi* is not even limited by the need to complete eclosion. Staggering the emergence period means that any adverse weather encountered in the summer months would not necessarily impede the species’ continued survival on the island, as posited by Hughes et al. (2013). The lack of mass emergence also suggests that *E. murphyi* has a more flexible life history, and emergence may be triggered by significant environmental cues for favourable conditions rather than any obligate physiology.

Parthenogenetic reproduction is a common feature within the Chironomidae, and in the Orthocladiinae usually takes the complete form of parthenogenesis known as thelytoky (Moller Pillot 2014; Scholl 1956; Thienemann 1954). In thelytoky, genetic fertilisation is absent and so females only produce female progeny, as is seen in *E. murphyi* (Convey 1992; Cranston 1985). It is likely that *E. murphyi* exhibits apomictic thelytoky, the most widespread from of thelytoky in Orthocladiinae (Scholl 1956, 1960) and that the lack of progression of a significant proportion of eggs beyond the initial development stage seen here is the result of a mechanical failing at an early maturation stage of mitosis (Porter and Martin 2011), possibly as the result of an environmental stressor. It is thought that the adoption of thelytoky by arthropods is an advantageous strategy. It may particularly benefit polar species, through the elimination of males, which have been shown to be more susceptible to the cold and extremes in temperature (Oliver and Danks 1972; Rinehart et al. 2000; David et al. 2005; Colinet and Hance 2009). Thus, the need for synchronous mass emergence is redundant. (Downes 1962; Porter and Martin 2011).

### Egg development

A total of 740 individual eggs were studied to document embryogenesis and hatching. Unusually for the sub-family Orthocladiinae, eggs are laid in an almost uniform spherical mass rather than in a rope-like mass or bale, a trait that is otherwise used to define the group (Nolte 1993) and that is also exhibited by *B. antarctica*. Individual egg morphology is consistent with previous descriptions of Orthocladiinae (Armitage et al. 1995; Nolte 1993; Thienemann and Strenzke 1940), and particularly that of *B. antarctica* (Harada et al. 2014). *Eretmoptera murphyi* individuals produced an average clutch size of 48 eggs in this study, of which only 29% hatched successfully. This is a small clutch size for a chironomid midge, which typically produce hundreds if not thousands of eggs, but not unusual for terrestrial Orthocladiinae which have been recorded to lay eggs in numerous small batches or even individually (Nolte 1993). Another Antarctic chironomid, *P. steinenii* lays an average of 191 eggs per batch, sometimes over multiple batches (Hahn and Reinhardt 2006), whilst Harada et al. (2014) describe a mean batch size of 41 eggs per string for *B. antarctica* with a gestation of just 16 days. Neither of these studies documented the percentage of eggs that go on to hatch.

With ground surface temperature variation of as much as 24.8 °C (see Online Resource 2) in a 12-h period on Signy Island, temperature stress may account for the long egg development time and high mortality observed. Despite the low clutch size and hatching success, our data indicate that at least 13 eggs will hatch for every *E. murphyi* adult. With population densities as high as 150,000 ind. m^2^ (Hughes and Worland 2010), this could result in as many as 1.2 million eggs being laid per m^2^ in parts of the species’ current distribution and, consequently, further local dispersal if no checks are held on the population at other points in the life cycle. However, as nearly half of all pupae failed to eclose to adult under field conditions, and 55% of adults failed to successfully oviposit, these two life stages appear to represent a major limitation. Whether this indicates lower stress tolerance in these life stages requires further study, given that only larval stages have been studied in detail to date. Even taking this into account, with an average density of *E. murphyi* within its distribution on Signy Island of 21,000 ind. m^2^ (Hughes and Worland 2010), we estimate that the species could have the potential to double its population over every life cycle of two years. If so, a simple back-calculation would suggest an introduction date of a single individual in the early 1970s, which is generally consistent with the assumed introduction event with plant transplants in the latter part of the 1960s (Burn 1982; Block et al. 1984).

### Phenology

Although all larval instars were present throughout the study period (Fig. 6b), their relative densities were highly variable over time. These data appear to reflect more the patchiness of larval distribution than the sequential occurrence of particular instars over time. Whilst eggs are immobile and thus easier to represent through repeated sampling in a fixed location, larvae are mobile. Patchiness in larval distribution, including aggregations of larvae, was also reported by Hughes and Worland (2010), and is a characteristic of Antarctic terrestrial invertebrate communities (Usher and Booth 1984). Any increase in hatched eggs naturally infers an increase in the number of L1 in the soil but, due to the very small size of this instar, it was not practicable to include them in this survey. Unhatched eggs do not overwinter. *Belgica antarctica* is also thought to overwinter only in the larval stage and in all four instars (Sugg et al. 1983). However, during soil core analyses, L1 and L3 *E. murphyi* larvae were noted to be molting towards the end of the season, indicating that L2 and L4 are likely to be the primary overwintering larval instars, as suggested by Convey (1996a) and Hughes et al. (2013).

**Fig. 7:**
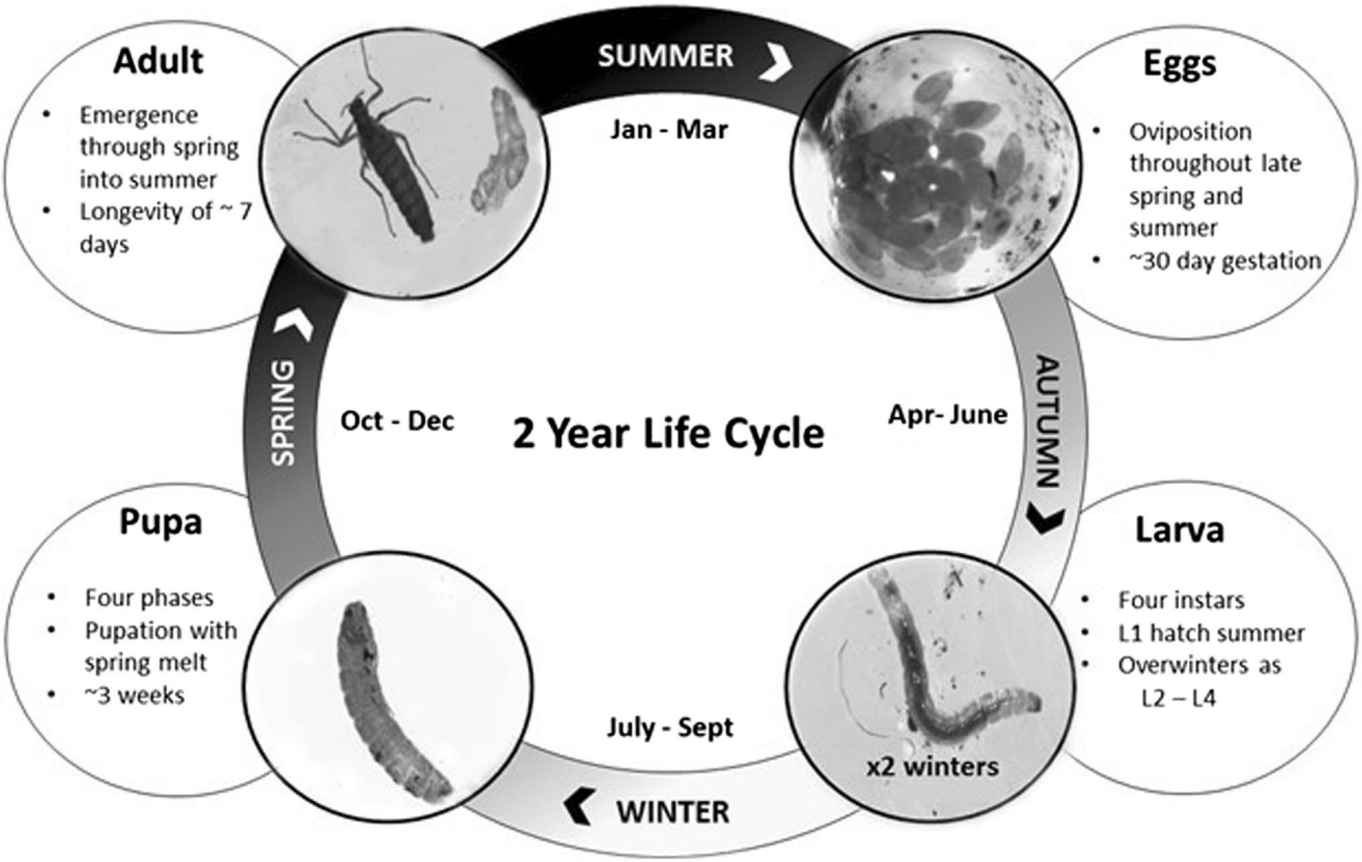
Summary of *Eretmoptera murphyi*’s life cycle

A mid-range climate forecast for the Antarctic Peninsula and South Orkney Islands suggests that mean annual air temperatures are expected to increase by 1.5–2 °C by 2100 (Larsen et al. 2014). Temperature warming is thought to benefit polar terrestrial invertebrates by reducing the stress of low-temperature extremes and giving greater liquid water availability (Bale and Hayward 2010; Convey 2006, 2011; Convey et al. 2014) and, in the case of non-native species, making available locations that were previously uninhabitable. By increasing our knowledge of *E. murphyi*’s life cycle, we can better understand any threat it may pose to Signy Island terrestrial ecosystems, and its potential as an invasive invertebrate at other at-risk areas, such as along the Antarctic Peninsula (Fig. 7).

## Conclusions

This study provides the first comprehensive documentation of the life cycle of *E. murphyi*, a flightless chironomid midge that is currently expanding its distribution following anthropogenic introduction to Signy Island. The species’ reliance on parthenogenesis is confirmed and new information provided on the characteristics of all life stages, their development rates, the phenology of previously undescribed eggs and pupae and the emergence of adults. Adults do not show synchronised emergence, rather appearing throughout the first half of the summer season, which suggests a flexible life history strategy where emergence is not dependent on any discrete environmental cue. Ground temperature variability and spikes in field temperature may explain the long development time of eggs compared to previous laboratory studies, and their low percentage of successful maturation. Despite the limitations on survival at each of the life stages, the population is potentially able to double in size every life cycle/2 years, highlighting the ability of this species to further expand its population and distribution on Signy Island. This study provides a springboard for further description and physiological studies of all life stages of this species, which will increase our understanding of the risks it poses as a non-native species on Signy Island, and the potential to colonise new areas, if given opportunity.

## Electronic supplementary material

Below is the link to the electronic supplementary material.

Electronic supplementary material 1 (PDF 113 kb)

Electronic supplementary material 2 (PDF 67 kb)
