# Supplementary material for: Life cycle and phenology of an Antarctic invader: the flightless chironomid midge, Eretmoptera murphyi
Source: Polar Biol. 2018 Sep 29;42(1):115–30. doi: 10.1007/s00300-018-2403-5 (PMC6390884; doi:10.1007/s00300-018-2403-5)
Supplement: Supplementary file 1 — Electronic supplementary material 1 (PDF 113 kb) [file 300_2018_2403_MOESM1_ESM.pdf]

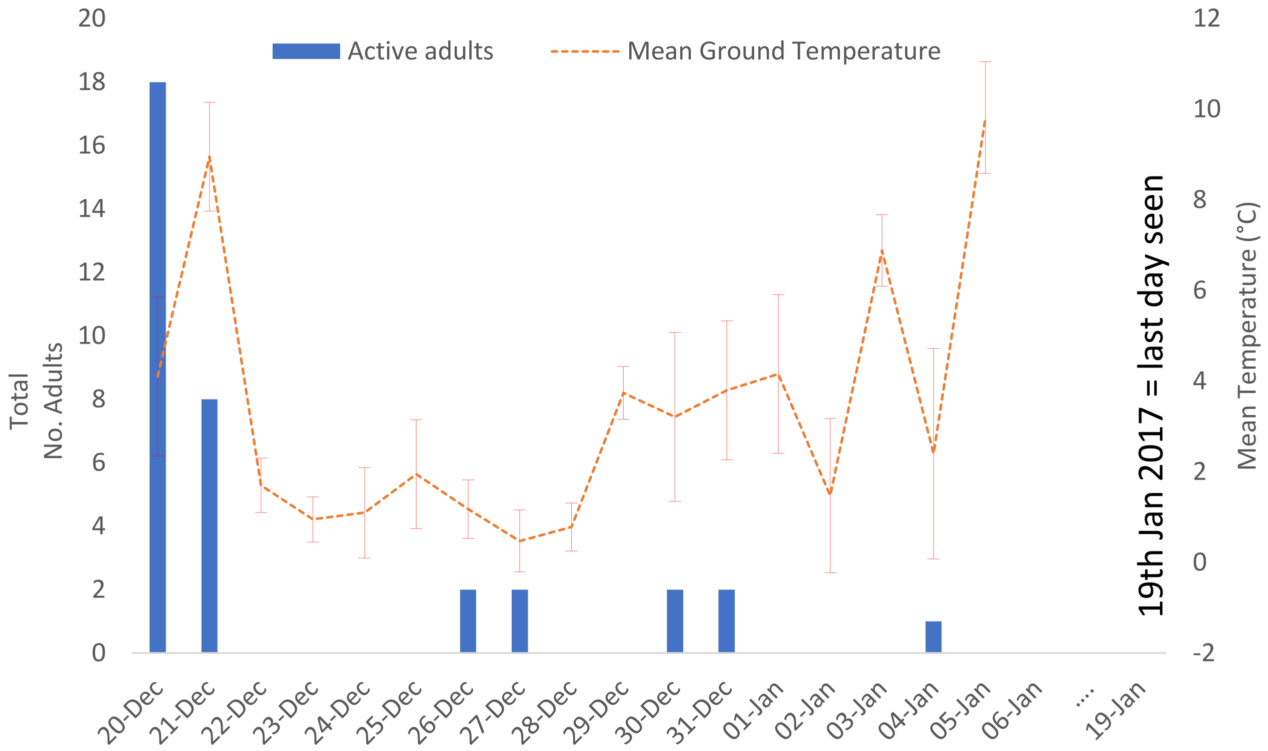

Online Resource 1 Emergence of adults within daily assessed quadrats, on Signy Island from 20th December 2016 until 6th January 2017, when populations had diminished. Plotted alongside mean (±SEM) ground temperature. Last adult seen active (not part of quadrat assessments) on 19th January 2017.
