# Supplementary material for: Life cycle and phenology of an Antarctic invader: the flightless chironomid midge, Eretmoptera murphyi
Source: Polar Biol. 2018 Sep 29;42(1):115–30. doi: 10.1007/s00300-018-2403-5 (PMC6390884; doi:10.1007/s00300-018-2403-5)
Supplement: Supplementary file 2 — Electronic supplementary material 2 (PDF 67 kb) [file 300_2018_2403_MOESM2_ESM.pdf]

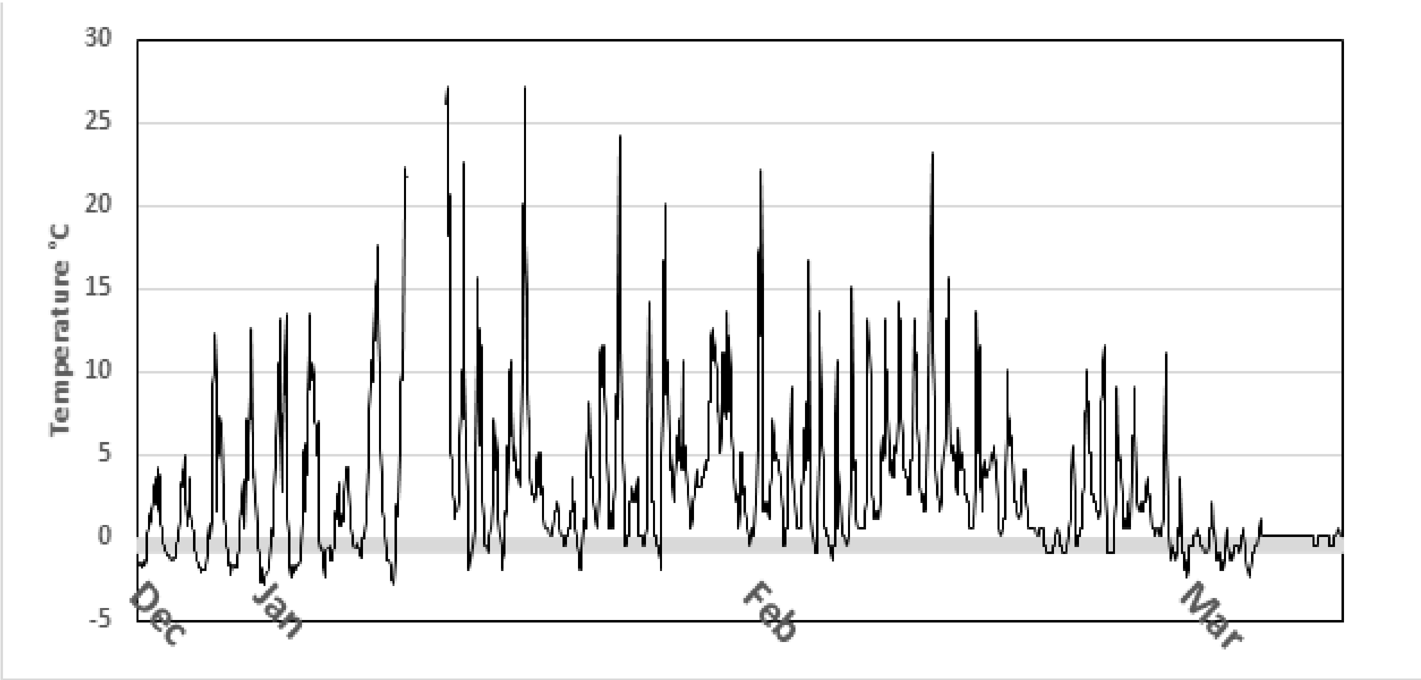

Online Resource 2 Ground surface temperatures recorded on the Signy backslope between the 28th December 2016 and the 10th March 2017. Temperatures logged every 30 minutes. Data from the 6th -12th January missing due to equipment removal by skua. Notably, the diurnal fluctuation on the ground is large, with values on the 5th January spanning from -2.8 at 5am to +22.6 at 3pm. Maximum temperature reached is 27.1 on the 12th January, minimum temperature was -2.8 on the 5th January..
